# Comparative Experiment of Abrasion-Corrosion-Sliding Wear Performance of Two Kinds of Low Alloy Wear-Resistant Steel

**DOI:** 10.3390/ma15186463

**Published:** 2022-09-17

**Authors:** Jinrong Chai, Guohua Li

**Affiliations:** 1 School of Mechanical Electronic and Information Engineering, China University of Mining and Technology (Beijing), Beijing 100083, China; 2 CCTEG Clean Energy Co., Ltd., Beijing 100013, China

**Keywords:** high-titanium low alloy wear-resistant steel, abrasive wear, corrosive wear, sliding friction and wear

## Abstract

There is a serious wear problem in the middle plate of scraper conveyors, which causes the problems of high transportation cost, low efficiency, and a lot of material waste. Therefore, it is necessary to study the wear performance of middle plate materials. A new high-titanium low alloy wear-resistant steel (ZM4-13) and a typical material (NM400) for middle plates are studied in this paper. The findings show that the mass loss of ZM4-13 and NM400 rises with the increase of coal gangue percentage. They do not increase monotonically with the change of pH value, and there is a critical value: the critical value of NM400 is between 6–8, and the critical value of ZM4-13 is between 7–9. When the pH value is less than the critical value, the mass loss decreases with the increase of pH value; when the pH value is greater than the critical value, the mass loss increases with the increase of pH value. Under the condition of high gangue and neutral solutions, ZM4-13 has better wear resistance. Its wear resistance can reach up to 1.09–2.10 times compared with NM400. The in-situ precipitated TiC particles are dispersed in ZM4-13. The high hardness of the TiC precipitation area in ZM4-13 hinders the plowing of hard particles and the plastic deformation of surface materials, so ZM4-13 is more wear-resistant than NM400, especially suitable for the harsh working conditions of coal mine production.

## 1. Introduction 

Coal machinery works in complex environments with corrosion, moisture, material particles, and dust, which causes serious wear [1,2,3,4,5]. According to incomplete statistics from relevant authorities, the economic losses caused by wear and tear in China’s coal mining system are estimated at more than 40 billion RMB each year [6]. The relative humidity in the underground coal mine can reach more than 95%, which makes the scraper conveyor stay in the high humidity environment for a long time, and some of them even work half-immersed in the mine water [7,8,9]. The wear of the middle plate, which is the key component of the scraper conveyor, is particularly significant [10]. 

At present, the commonly used materials for the medium plate in China are NM400, HARDOX450, and JF500, among which NM400 has become one of the most commonly employed materials for medium plates due to its high cost performance [11]. In recent years, the friction and wear properties of the middle plate have been studied. Zhiyuan Shi et al. [12] established the distribution diagram of wear mechanism of NM400 under severe working conditions. Ilyas Hacısalihoğlu et al. [13] investigated the tribocorrosion behavior of plasma nitrided HARDOX steels in NaCl solution. Junxia Li et al. [5] carried out some experiments under dry wear conditions using middle plate material. Chengru Li et al. [14] investigated sliding wear behavior of NM400 against silicon nitride within the temperature range of 300–500 °C. Recently, low alloy wear-resisting steels containing Ti have attracted attention because of their high wear-resistant properties [15,16]. The wear resistance of low alloy wear-resistant steels containing Ti can reach up to 1.3 times under dry wear conditions compared with Hardox450 [15] and 1.4 times under wet wear conditions compared with NM400 [16]. 

Limited by wear testing equipment, a rubber wheel is used in three-body abrasive wear research of medium plate, which is different from the actual working conditions of a medium plate, and the research results may have errors in guiding the material selection of medium plate. Therefore, it is necessary to study the wear performance of medium plate materials in the actual working conditions. A novel sliding friction and wear tester has been developed which can better simulate the actual conditions of the middle plate [17]. In this paper, the wear behavior and wear mechanism of ZM4-13 and NM400 are investigated under complex working conditions by the novel sliding friction and wear tester. The research results can provide important reference data for the research, development, and application of middle plate materials.

## 2. Test Scheme

### 2.1. Test Materials

#### 2.1.1. Materials of Samples and Characterization Methods

**Figure 1:**
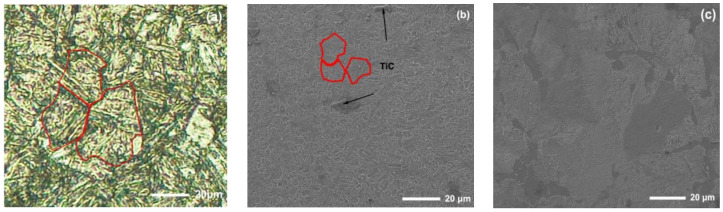
Microstructure of the test materials: (**a**) NM400; (**b**) ZM4-13; (**c**) 40Cr.

**Figure 2:**
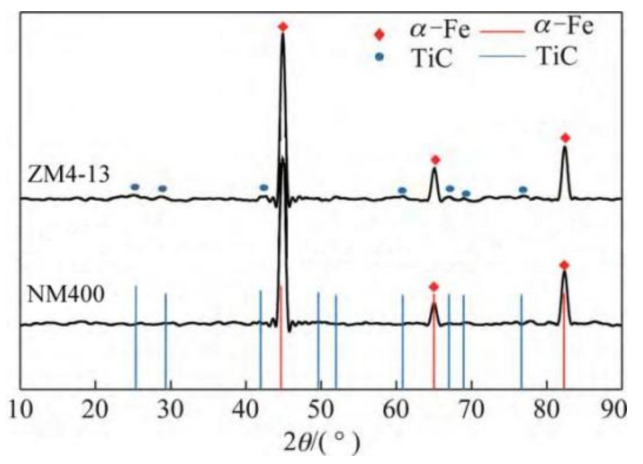
XRD patterns of samples and standard X-ray diffraction peaks of α-Fe and TiC.

The new middle plate material ZM4-13 and the common middle plate material NM400 were selected for the sample, and the upper sample was 40Cr steel. 40Cr steel is the common steel that is used for the chain of scraper conveyors. The actual friction pair is the middle plate and the chain. In order to simulate the actual working conditions better, the material with similar hardness to the chain was selected for the upper sample. The chemical compositions of the samples are shown in Table 1. Rockwell hardness (HRC) tests of ZM4-13 and NM400 were carried out on a TH930 digital Brinell hardness tester. The test load was 1471 N and the loading time was 15 s. To ensure the accuracy and reliability of the data, five points were tested in different areas of each sample, and then the average value was taken as the final hardness data. The average hardness of ZM4-13, NM400, and 40Cr is 43.94 ± 0.63, 38.04 ± 0.52, and 34.36 ± 0.57 HRC, respectively. The metallographic structure of the materials were observed and analyzed under ICX41M inverted metallographic microscope. The metallographic structure of the material is shown in Figure 1. The metallographic structure of ZM4-13 and NM400 is lath martensite, and the grain size of ZM4-13 is smaller than that of NM400. The metallographic structure of 40Cr is lamellar pearlite and ferrite. The phase structure was analyzed using a Panalytical Aeris X-ray diffractometer. Cu target material Kα radiation was employed, and the tube current and tube voltage were 15 mA and 40 kV, respectively. The XRD analysis results are shown in Figure 2. The red line in the figure represents the standard X-ray diffraction peak of α-Fe, while the blue line represents the standard diffraction peak of TiC. The phase structure of ZM4-13 is α-Fe, and so is NM400. ZM4-13 contains TiC particles. TiC particles act as nails on the austenite grain boundary and can prevent the austenite from growing. In addition, because Ti and C form TiC, the solid solubility of C in the solid solution decreases, so the effect of carbon on the bonding force of γ-Fe lattices is weakened, and the growth of austenite grains is further prevented. The Ti element plays the role of refining grain.

#### 2.1.2. Preparation of Abrasive

**Figure 3:**
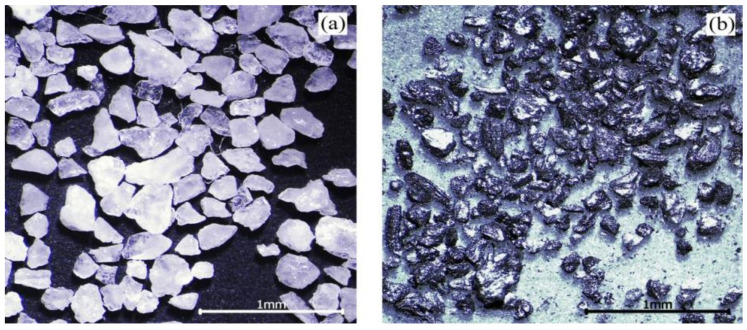
Morphology of the abrasives: (**a**) quartz sand; (**b**) cleaned coal [17].

In actual working conditions, raw coal contains coal and coal gangue. Coal gangue is not suitable for using as a test abrasive. Instead of coal gangue, quartz sand was mixed with cleaned coal to make an abrasive, due to its mature technology and controllable particle size and hardness. The proportion of coal and quartz sand was expressed by the percentage of coal gangue. For example, if the percentage of coal gangue is 30%, then there is 70 g coal and 30 g quartz sand in 100 g abrasive. The cleaned coal is anthracite produced in Shanxi. The particle size of cleaned coal and quartz sand is 60–80 mesh. The abrasive morphology is shown in Figure 3.

#### 2.1.3. Preparation of Corrosion Solution

The corrosion solution was simulated mine water, which is composed of CaSO_4_, MgSO_4_, Na_2_SO_4_, KCl, NaCl, CaCl, H_2_SO_4_ (C (H^+^) = 0.2 mol/L), NaOH (C (OH^+^) = 0.1 mol/L), and distilled water. The content of each substance in the simulated mine water is shown in Table 2.

### 2.2. Wear Tester

#### 2.2.1. Overall Structure and Working Principle

**Figure 4:**
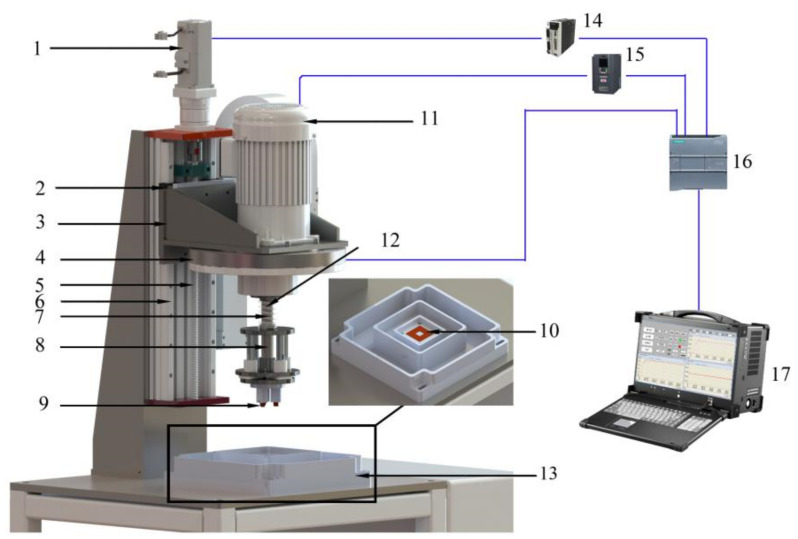
Schematic diagram of the friction and wear tester that can simulate the complex working conditions of a coal mine [17]: (1) Servo motor, (2) Slider, (3) Loading flange, (4) Positive-pressure detection mechanism, (5) Screw, (6) Linear guide rail, (7) Spring, (8) Grinding head mechanism, (9) Upper samples, (10) Samples, (11) Frequency conversion motor, (12) Spindle, (13) Corrosion chamber, (14) Servo driver, (15) Frequency converter, (16) Programmable logic controller, (17) Principal computer.

The sliding friction and wear tester for under complex working conditions can carry out a variety of friction and wear tests. A structural diagram of the tester is illustrated in Figure 4 [17]. As shown in the figure, the tester is composed of four parts: an automatic loading mechanism, an automatic speed regulating mechanism, a corrosion tank, and a control display system. The automatic loading mechanism is composed of a servo motor (1), slider (2), loading flange (3), positive-pressure detection mechanism (4), screw (5), linear guide rail (6), spring (7), grinding head mechanism (8), upper samples (9), and samples (10). The automatic speed regulating mechanism is composed of a frequency conversion motor (11) and spindle (12). The corrosion chamber (13) contains a corrosion table, an abrasive chamber, a corrosive liquid chamber, and an overflow chamber. The control display system consists of an electrical control system and an upper computer display system. Its main components include a servo driver (14), frequency converter (15), programmable logic controller (PLC) (16), and principal computer (17). The loading accuracy of the automatic loading mechanism is ±1 N, and that of the automatic speed-regulating mechanism is ±1 rpm.

#### 2.2.2. Sample Structure

**Figure 5:**
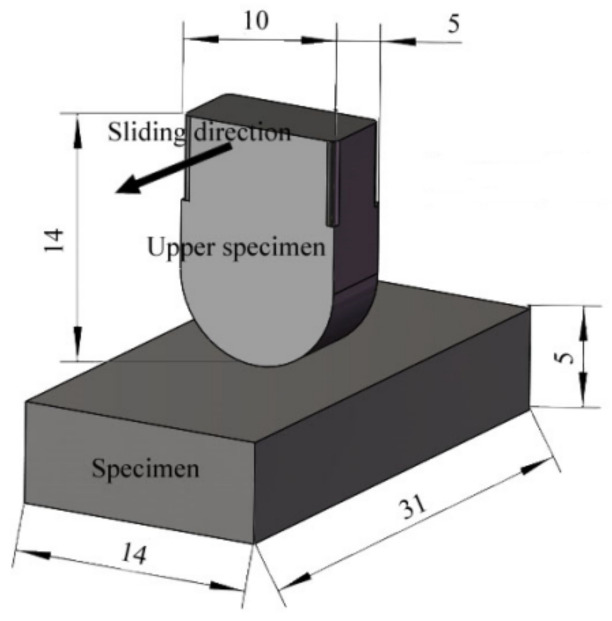
Schematic diagram of sample structure [17].

In order to simulate the actual contact form between the middle plate and the chain, the upper sample is designed as a curved block structure with a length of 14 mm, a width of 10 mm, and a thickness of 5 mm. The curved bottom makes the abrasive enter the friction and wear interface easily. The sample is designed as a block structure, with a length of 31 mm, a width of 14 mm, and a thickness of 5 mm, as shown in Figure 5.

### 2.3. Test Conditions and Process

The tests were carried out at room temperature under the conditions of 150 N load, 100 rpm speed, different coal gangue contents (30%, 40%, 50%, 60%, and 70%), and different mine drainage pH values (3.5, 5.5, 7, 9.5, and 11.5). Each test lasted for 60 min, and the samples were cleaned and dried with alcohol before and after each test. Samples were weighed on an electronic balance with an accuracy of 0.1 mg to calculate their wear mass loss. Each test was repeated three times and the average of mass loss was taken as the test result. A JCM-7000 scanning electron microscope (SEM) was used to analyze the microstructure and the distribution of the element of the worn surface. The SEM analysis conditions were 15 kV voltage, a working distance (WD) of 12.5 mm.

## 3. Test Results and Analysis

### 3.1. The Effect of Test Conditions

**Figure 6:**
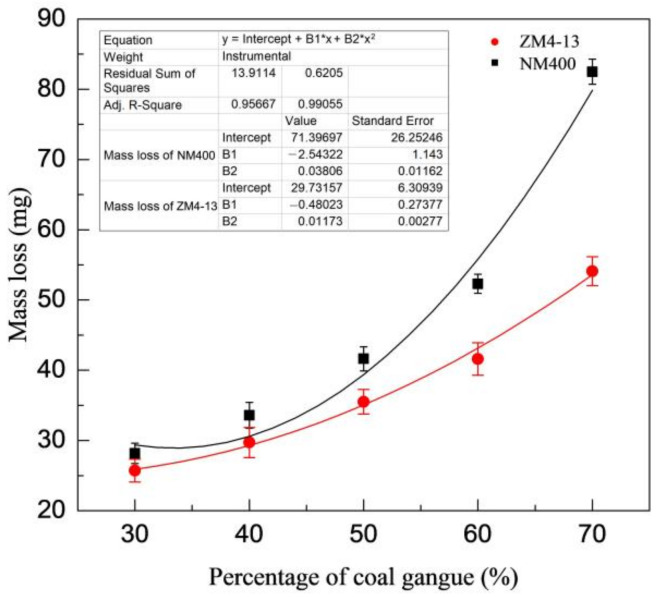
Change of the mass loss with the percentage of coal gangue.

**Figure 7:**
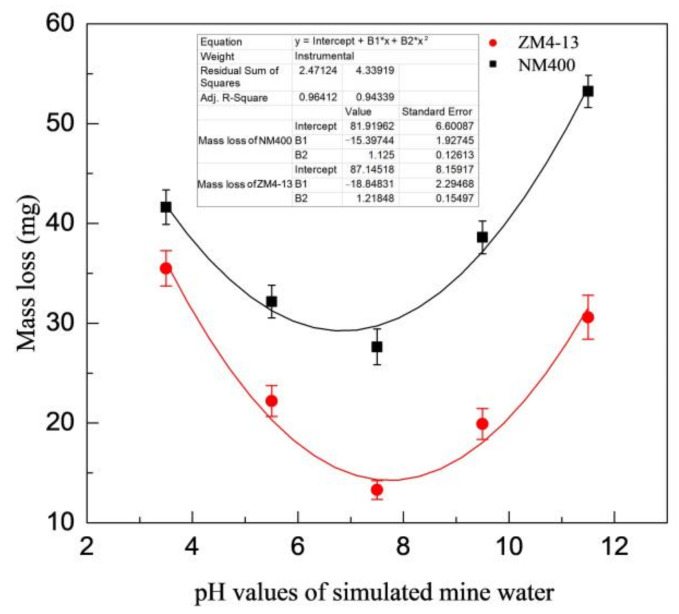
Change of the mass loss with pH value of simulation mine water.

Figure 6 shows the change of mass loss of ZM4-13 and NM400 with the percentage of coal gangue. As shown in the figure, the mass loss of NM400 first increases slowly with the percentage increase of coal gangue. When the percentage of coal gangue exceeds 50%, the mass loss increases rapidly. The wear of ZM4-13 increases with the increase of coal gangue content, and it does not show a turning point of rapid increase. Figure 7 shows the change of mass loss of ZM4-13 and NM400 with the pH value of simulated coal mine water. The mass loss of NM400 and ZM4-13 does not increase monotonically with the pH value, but critical values are observed. The critical value of pH for NM400 is between 6 and 8, and that for ZM4-13 is between 7 and 9. When the pH value is less than the critical value, the mass loss decreases with the increase of pH value, and when the pH value is greater than the critical value, the mass loss increases with the increase of pH value.

The anti-wear effect is expressed by the relative wear-resistant coefficient ε, whose calculation formula is as follows [18]:

$$\varepsilon =\frac{\Delta X_{1}/\rho_{1}}{\Delta X_{2}/\rho_{2}}$$

where Δ*X*_1_ and Δ*X*_2_ are the mass loss of NM400 and ZM4-13, respectively. *ρ*_1_ and *ρ*_2_ are the densities of them, respectively. The densities of them are similar, i.e., *ρ*_1_ is approximately equal to *ρ*_2__._ The increase of ε indicates the increase of wear-resistance.

**Figure 8:**
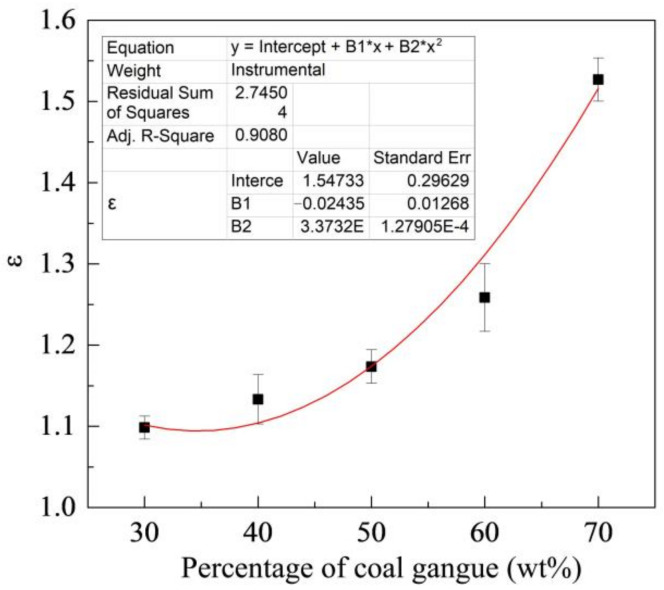
Change of relative wear-resistant coefficient with the percentage of coal gangue.

Figure 8 shows the change of ε with the percentage of coal gangue. As shown in the figure, the ε increases with the percentages of coal gangue. ZM4-13 is more wear-resistant than NM400 under different percentages of coal gangue and has better wear resistance under high gangue conditions. Its wear resistance can reach up to 1.52 times as the maximum, compared with NM400.

**Figure 9:**
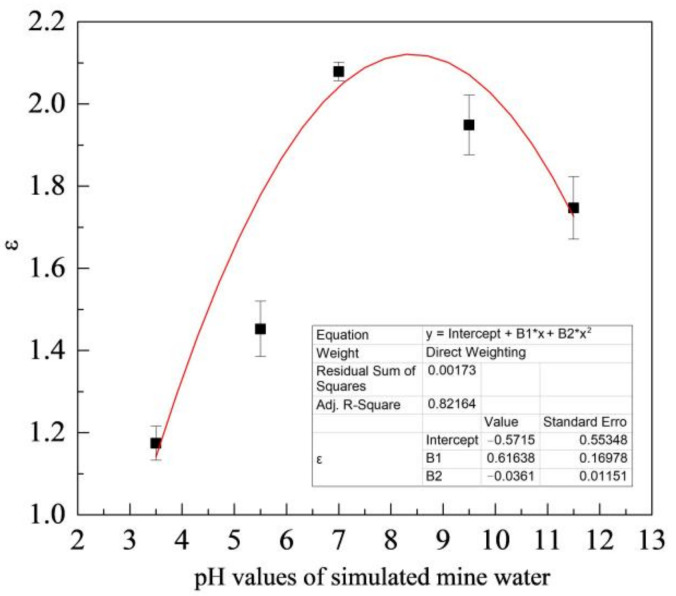
Change of relative wear-resisting coefficient with pH values of simulated mine water.

Figure 9 shows the variation of ε with the pH value of simulated coal mine water. As shown in the figure, the mass loss of ZM4-13 is smaller than that of NM400, ZM4-13 is more wear-resistant than NM400 and has better wear resistance in neutral solution. Its wear resistance can reach up to 2.1 times as the maximum, compared with NM400.

### 3.2. Wear Mechanism

**Figure 10:**
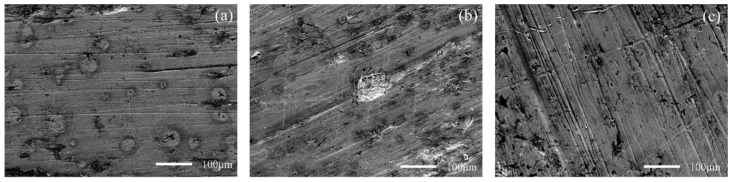
SEM morphology of worn surfaces of ZM4-13 with varying percentage of coal gangue at acid simulated mine water: 30 wt% (**a**), 50 wt% (**b**), 70 wt% (**c**).

**Figure 11:**
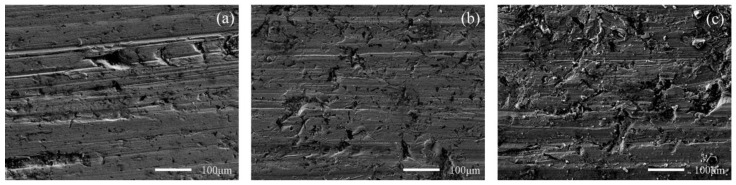
SEM morphology of worn surfaces of NM400 with varying percentage of coal gangue at acid simulated mine water: 30 wt% (**a**), 50 wt% (**b**), 70 wt% (**c**).

Figure 10 and Figure 11 show the worn surface of NM400 and ZM4-13, respectively, which had undergone a period of 60 minutes with the varied percentage of coal gangue at acid simulated mine water. As shown in Figure 10a and Figure 11a, the worn surfaces of ZM4-13 and NM400 at the percentage of coal gangue of 30% showed signs of slight scratches and corrosion pits, and there are also signs of shedding pits on the worn surface of NM400. The plowing effect of hard particles on the surface of the material continuously formed a clean surface. These clean surfaces were constantly corroded, and formed chemicals. These substances were continually removed from the surface by microscopic cutting of hard particles. Because of the low percentage of hard particles in the abrasive, the interaction between abrasive wear and corrosion wear was weak. The main wear of ZM4-13 and NM400 are abrasive wear and corrosion wear at this percentage of coal gangue. As shown in Figure 10b and Figure 11b, the worn surfaces of ZM4-13 and NM400 at the percentage of coal gangue of 50% showed signs of more scratches and corrosion pits, and there are also signs of plastic deformation on the worn surface of NM400. This is caused by the increase of hard abrasive content, which on the one hand, aggravates the abrasive wear and corrosion wear mutually promoted; on the other hand, a clean surface also easily causes adhesive wear, such as NM400 after the grinding surface appeared with obvious plastic deformation. At this percentage of coal gangue, the main wear on ZM4-13 was abrasive wear and corrosion wear, and the main wear on NM400 was abrasive wear, corrosion wear and adhesive wear. As shown in Figure 10c and Figure 11c, when the percentage of coal gangue was 70%, more scratches and corrosion pits appeared on the worn surface of ZM4-13 and NM400, and the plastic deformation on the worn surface of NM400 was more serious; the corrosion pits and peeling pits were connected. With the increase of coal gangue percentage, the abrasive wear was aggravated, and the corrosion wear and adhesive wear were further promoted. Under the combined action of abrasive wear, corrosion wear and adhesive wear, ZM4-13 and NM400 wore more seriously. However, the degree of adhesive wear of ZM4-13 was weak, and the wear of ZM4-13 was mainly abrasive wear and corrosion wear, while the wear of NM400 was mainly abrasive wear, corrosion wear, and adhesive wear. 

**Figure 12:**
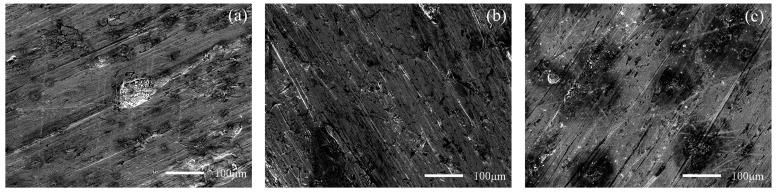
SEM morphology of worn surfaces of ZM4-13 with varying pH values of simulated mine water at the percentage of coal gangue of 50%: 3.5 (**a**), 7 (**b**), 11.5 (**c**).

**Figure 13:**
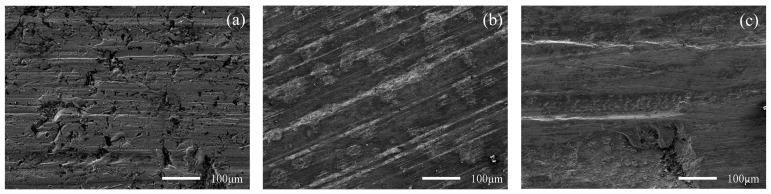
SEM morphology of worn surfaces of NM400 with varying pH values of simulated mine water at the percentage of coal gangue of 50%: 3.5 (**a**), 7 (**b**), 11.5 (**c**).

**Figure 14:**
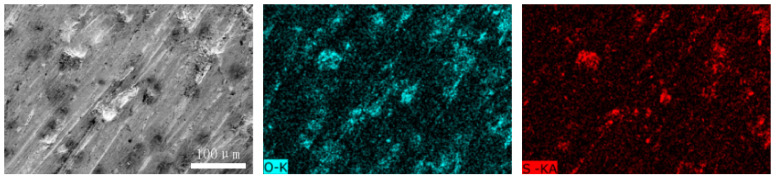
The element distribution of worn surface of ZM4-13 under coal gangue percentage of 50% and pH values of simulated mine water of 3.5.

**Figure 15:**
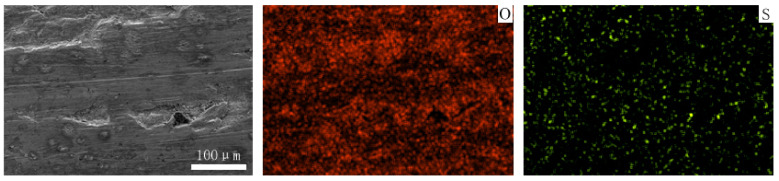
The element distribution of worn surface of NM400 under coal gangue percentage of 50% and pH values of simulated mine water of 3.5.

**Figure 16:**
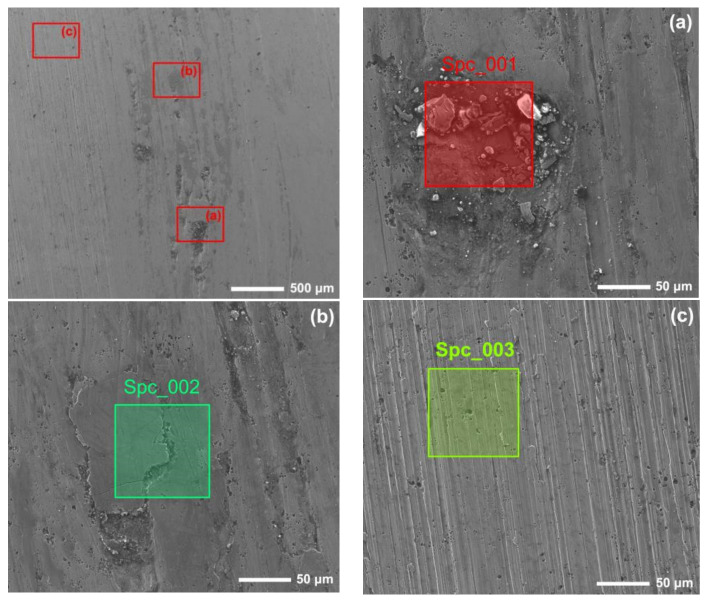
The energy spectrum analysis of worn surface of ZM4-13 under coal gangue percentage of 50% and pH values of simulated mine water of 3.5: worn and corrosive zone 1 (**a**), worn and corrosive zone 2 (**b**), corrosive zone (**c**).

**Figure 17:**
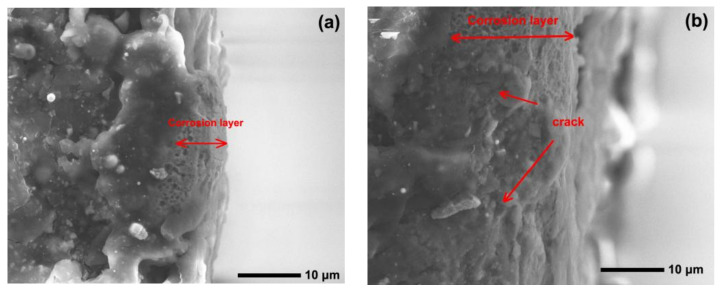
Cross-section SEM of ZM4-13 under coal gangue percentage of 50% and pH values of simulated mine water of 3.5: corrosive zone (**a**), worn and corrosive zone (**b**).

Figure 12 and Figure 13 show the worn surfaces of NM400 and ZM4-13, respectively, which had undergone a period of 60 min with the varied pH values of simulated mine water at the percentage of coal gangue of 50 wt%. As shown in Figure 12a and Figure 13a, the worn surfaces of ZM4-13 at pH values of simulated mine of 3.5 showed the signs of scratches, slight plastic deformation and corrosion pits, and obvious scratches, more plastic deformation and corrosion pits on the worn surface of NM400. NM400 was more seriously worn than ZM4-13. Under the action of certain positive pressure and micro-cutting of hard particles, the material surface produces plastic deformation, which promotes the diffusion of O, S and other elements in the corrosion solution to the deformation layer (as shown in Figure 14 and Figure 15). The energy spectrum analyses of worn surface of ZM4-13 were shown in Figure 16 and Table 3. Comparing the corrosion wear zone and the corrosion zone, it is found that the oxygen element in the corrosion wear zone is higher than that in the corrosion wear zone, indicating that there is oxide formation in the corrosion wear zone. The presence of sulfur in the corrosion-wear zone indicates the formation of sulfide. The carbon element in the corrosion wear zone 1 and the corrosion zone is much higher than that in the material itself, which may be because the coal in the abrasive remains in the surface damage zone. The elements Al, K, and Ca in the corrosion wear zone are derived from mine water solution. Cross-section SEM of ZM4-13 was shown in Figure 17. The worn and corrosive zone is more severe than the corrosive zone, and cracks appear in the subcutaneous layer. This indicates that wear can accelerate corrosion. On the worn surface, hard and brittle oxides and soft sulfides are constantly formed on the worn surface and fall off from the surface under the micro-cutting action of hard particles. This cycle leads to material loss. The main wear of ZM4-13 and NM400 is abrasive wear and corrosion wear. As shown in Figure 12b and Figure 13b, the worn surfaces of ZM4-13 and NM400 at pH values of simulated mine of 7 showed the signs of slight scratches. The wear is lighter than in acidic conditions. This is because neutral mine water is not conducive to the diffusion of O, S, and other elements to the material deformation layer, the mutual promotion between plastic deformation and element diffusion is weakened, and the material loss is reduced. The wear of ZM4-13 and NM400 is mainly abrasive wear. As shown in Figure 12c and Figure 13c, the worn surface of ZM4-13 at pH values of simulated mine of 11.5 showed the signs of slight scratches, the same as neutral condition, but NM400 was worn more severely, and there are large area losses on the worn surface. Alkaline mine water contributes to the diffusion of O, S, and other elements to a certain extent, but this promoting effect is less than that of acid mine water. The main wear of ZM4-13 and NM400 is abrasive wear and corrosion wear. 

### 3.3. Anti-Wear Mechanism of ZM4-13

**Figure 18:**
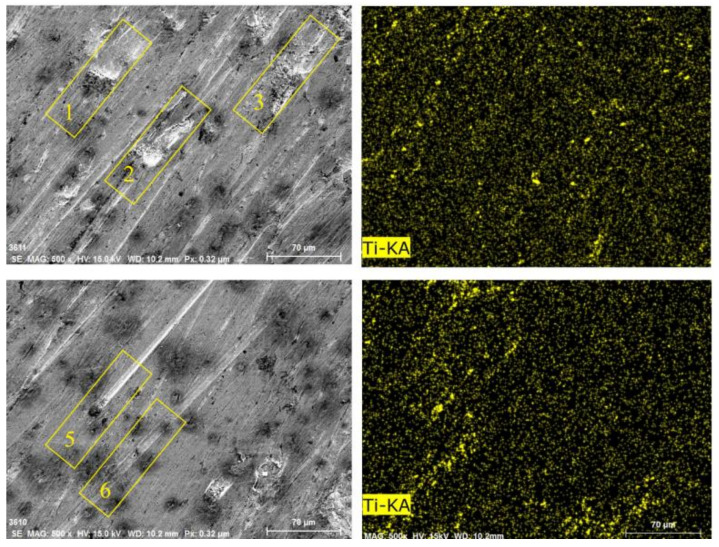
Surface morphologies and Ti surface distribution of ZM4-13 after worn under coal gangue percentage of 50% and simulated mine water of 3.5.

Figure 18 shows the Ti distribution on the worn surface of ZM4-13. There are some approximately circular pits on the surface of ZM4-13, compared to the long strip pits on the worn surface of NM400. The TiC precipitation region has higher hardness than martensite matrix which impedes the plowing of hard particles and adhesive wear. This behavior can be clearly observed in areas 1, 2, and 3 in Figure 16. When the abrasive particles act on the surface of ZM4-13, the material is more easily pushed to both sides without breaking, forming a furrow. When subjected to subsequent abrasive action, the accumulated material on either side of the furrow may be re-flattened, or the deformed material may be subjected to another furrow deformation [7]. There is no obvious furrow in the Ti-concentrated area. The furrow in Zone 5 of Figure 16 was blocked by the Ti-concentrated area, and the furrow in Zone 6 becomes shallow after passing through the Ti-concentrated area. This indicates that the TiC precipitation region hinders the plowing effect of the abrasive. Thus, ZM4-13 is more wear-resistant.

## 4. Conclusions

The abrasive-corrosion-sliding friction and wear behavior of ZM4-13 and NM400 were studied by a novel sliding friction and wear tester. The specific conclusions are as follows:

(1) The mass loss of ZM4-13 and NM400 increases with the increase of the percentage of coal gangue. They don’t increase monotonically with the change of pH value, and there is a critical value: the critical value of NM400 is between 6–8, and the critical value of ZM4-13 is between 7–9. When the pH value is less than the critical value, the mass loss decreases with the increase of pH value; when the pH value is greater than the critical value, the mass loss increases with the increase of pH value. The main wear of ZM4-13 and NM400 is abrasive wear and corrosion wear in acidic and alkaline mine water, and abrasive wear in neutral mine water.
(2) ZM4-13 steel is more wear-resistant than NM400 under different gangue percentages and pH values of mine water, and has better wear resistance under high gangue conditions and neutral mine water. The wear resistance of ZM4-13 is 1.09–2.10 times, compared with NM400.
(3) The high hardness of TiC precipitation area in ZM4-13 hinders the plowing of hard particles and the plastic deformation, so ZM4-13 is more wear-resistant than NM400, and is especially suitable for the harsh working conditions of coal mine production.

## Figures and Tables

**Table 1:** The chemical composition of experimental materials (wt.%).

Materials	C	Si	Mn	P	S	Ti	Cr	Mo	B	Ni	Cu	Al
ZM4-13	0.30–0.38	0.20–0.60	0.30–1.20	≤ 0.015	≤ 0.003	0.40–0.80	0.60–1.50	0.20–0.50	0.001–0.003	0.40–1.20	0.10–0.50	0.0015–0.065
NM400	0.160	0.230	1.420	≤ 0.015	≤ 0.003	0.012	0.170	0.020	0.001	-	-	-
40Cr	0.37–0.44	0.17–0.37	0.50–0.80	≤ 0.035	≤ 0.035	-	0.80–1.10	≤ 0.01	-	≤ 0.3	≤ 0.03	-

**Table 2:** Concentration of substances in the simulated mine water.

pH Value	CaSO_4_ (mg/L)	MgSO_4_ (mg/L)	Na_2_SO_4_ (mg/L)	KCl (mg/L)	NaCl (mg/L)	CaCl (mg/L)	H_2_SO_4_ (mL/L)	NaOH (mL/L)
3.5	70	50	40	12	141	10	40	-
5.5	70	50	40	12	141	10	22	-
7	70	50	40	12	141	10	-	-
9.5	70	50	40	12	141	10	-	32
11.5	70	50	40	12	141	10	-	100

**Table 3:** Element composition of spectrum (at. %).

No.	C	O	Al	Si	S	K	Ca	Ti	Cr	Mn	Fe	Ni	Total
Spc_001	48.30	28.79	0.47	1.01	0.23	0.57	0.29	0.12	0.40	0.20	19.42	0.21	100
Spc_002	17.02	48.55	-	0.28	0.41	0.47	-	0.11	0.36	-	32.81	-	100
Spc_003	26.90	3.41	0.32	0.49	-	-	-	0.67	0.82	0.49	66.91	-	100

## Data Availability

The study did not report any data.
